# Molecular Orientation-Induced Second-Harmonic Generation: Deciphering Different Contributions Apart

**DOI:** 10.1021/acs.jpca.2c03237

**Published:** 2022-06-02

**Authors:** Amit Beer, Ran Damari, Yun Chen, Sharly Fleischer

**Affiliations:** † Raymond and Beverly Sackler Faculty of Exact Sciences, School of Chemistry, Tel Aviv University, Tel Aviv 6997801, Israel; ‡ Tel-Aviv University Center for Light-Matter-Interaction, Tel Aviv 6997801, Israel

## Abstract

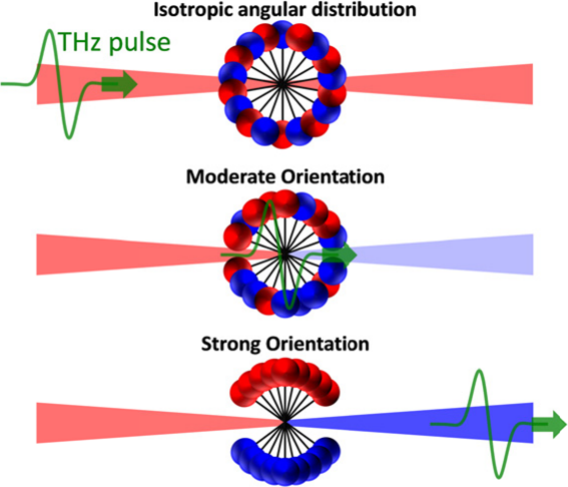

We demonstrate and explore an all-optical technique for direct monitoring of the orientation dynamics in gas-phase molecular ensembles. The technique termed “MOISH” utilizes the transiently lifted inversion symmetry of polar gas media and provides a sensitive and spatially localized probing of the second-harmonic generation signal that is directly correlated with the orientation of the gas. Our experimental results reveal selective electronic and nuclear dynamical contributions to the overall nonlinear optical signal and decipher them apart using the “reporter gas” approach. “MOISH” provides new crucial means for implementing advanced coherent rotational control via concerted excitation by both terahertz and optical fields.

## Introduction

Angular control of gas molecules is a long-standing goal of physics and chemistry, aimed to lift the inherent isotropy of gas molecules to aid the extraction of spectroscopic signatures from the molecular frame. Vast research efforts have successfully yielded a plethora of new observations and possibilities over the years, ranging from basic light-matter phenomena through novel spectroscopic methods for studying rotational dynamics to practical coherent control schemes and many more.^1−6^ Anisotropic angular distributions are categorized as “aligned” or “oriented”, referring to the preferable distribution of the *intramolecular axis* along a specific lab-frame axis or the *molecular dipoles* toward a specific lab-frame direction, respectively.^7,8^ Correspondingly, alignment retains the inversion symmetry of the medium, whereas orientation entails its inversion asymmetry upon orientation of the molecular dipoles toward the +*z* or −*z* direction (“up” or “down”).^9,10^ The lifted inversion symmetry provides access to nonlinear optical responses of even orders in the field ( ∝ *E*^2*n*^) that are otherwise forbidden in unordered gas samples. Orientation may be induced by two-color laser field,^11−14^ mixed field (dc + optical pulse),^15,16^ or terahertz (THz) field excitations^8,17−20^ that interact resonantly via the permanent molecular dipole. Furthermore, intense THz fields have shown to induce molecular alignment in liquid and gas phases via the terahertz Kerr effect that is detected via time-resolved optical birefringence measurements.^8,10,21−23^ We note that nonresonant near-infrared (NIR) pulses with twisted polarizations have recently shown to induce molecular orientation.^24,25^

THz fields induce molecular orientation by dipole interaction with polar molecules *V̂* = – μ⃗ · *E⃗* to create a rotational wavepacket , where  with *L̂* being the angular momentum operator,  the spherical harmonic functions, and their expansion coefficients *c*_*J*, *m*_. The rotational wavepacket ψ(*t*) periodically reproduces itself at integer multiples of the “rotational revival time”,^26,27^ given by  (*B* is the rotational constant of the molecule).

As the gas molecules periodically orient, they form a transient macroscopic dipole that manifests by the emission of THz bursts, usually referred to as free-induction signals (FIDs)^8,18,19,28^ detectable via time-resolved electro-optic sampling (EOS).^29,30^ EOS provides an indirect signature of orientation because the radiated FID signals follow the time-derivative of the orientation –d⟨cos θ⟩/d*t* rather than the orientation ⟨cos θ⟩ itself.^8,31^ The main obstacle imposed by EOS (and FID) as a probe of molecular orientation emanates from the fact that the FID signals are accumulated throughout the entire interaction volume of the THz field and the gas, thereby lacking any spatial resolution. While the latter enables rotational spectroscopic measurements, it compromises and even practically impedes advanced coherent control schemes. For example, FID signals that emanate from nonlinear THz excitation at the focus of the THz field are accompanied and practically embedded in large FID signals irradiated from the entire volume, where the THz amplitude is much lower and the signals are governed by the linear response to the field. The most relevant example for our research endeavors is the utilization of both NIR and THz fields in concert for advanced control of molecular dynamics. While an NIR pulse interacts with the molecules via the anisotropic polarizability tensor, THz fields interact through the permanent molecular dipoles, together providing two distinct rotational control handles.^19^ The extreme difference in volumetric foot-prints of the THz and NIR in the gas sample, with focal diameters of ∼3 mm and ∼50 μm, respectively, practically thwarts the detection of FID signals that selectively arise from molecules that experienced both the NIR and THz excitations. Motivated by the need for spatially localized detection of molecular orientation, we set to monitor the transient inversion asymmetry of the gas via the SHG (λ_s_ = 400 nm) of a NIR probe (λ_probe_ = 800 nm).

## Experimental Methods

The experimental approach presented hereafter is closely related to the THz field-induced SHG (TFISH)—a technique used for the detection of broad-band THz fields.^32−36^ TFISH relies on the nonlinear mixing of three input fields—a THz field (*E*_THz_) and two NIR fields (*E*_ω_, *E*_ω_) via the third-order susceptibility, χ^(3)^, to yield a signal field at frequency ω_TFISH_ ≅ 2ω. While TFISH is restricted to nonpolar gases^33^ and typically performed in ambient air, “MOISH” aims to probe the rotational dynamics of polar gas samples that are resonantly excited by THz fields and manifest transient orientation dynamics long after the THz field is over. Special efforts are made to decipher the electronic (TFISH) and nuclear orientation (MOISH) contributions apart at the fundamental times of the rotational evolution of polar gases.

The experimental setup used in this work is similar to that reported in ref (35). Briefly, intense THz and NIR probe beams are routed to propagate collinearly and focus inside a static gas cell equipped with a designated <1 mm aperture. The latter effectively restricts the interaction length of the two beams and eases the phase-mismatch (Δ*k*) of the generated SH signal. The NIR pulse (100 fs duration, 6 μJ pulse energy) is focused by a lens (*f* = 150 mm) such that its intensity remains well below the laser-induced plasma regime.^35,37,38^ Complementary EOS measurements were performed in our home-built time-domain THz spectrometer.^18,19^

## Results and Discussion

**Figure 1:**
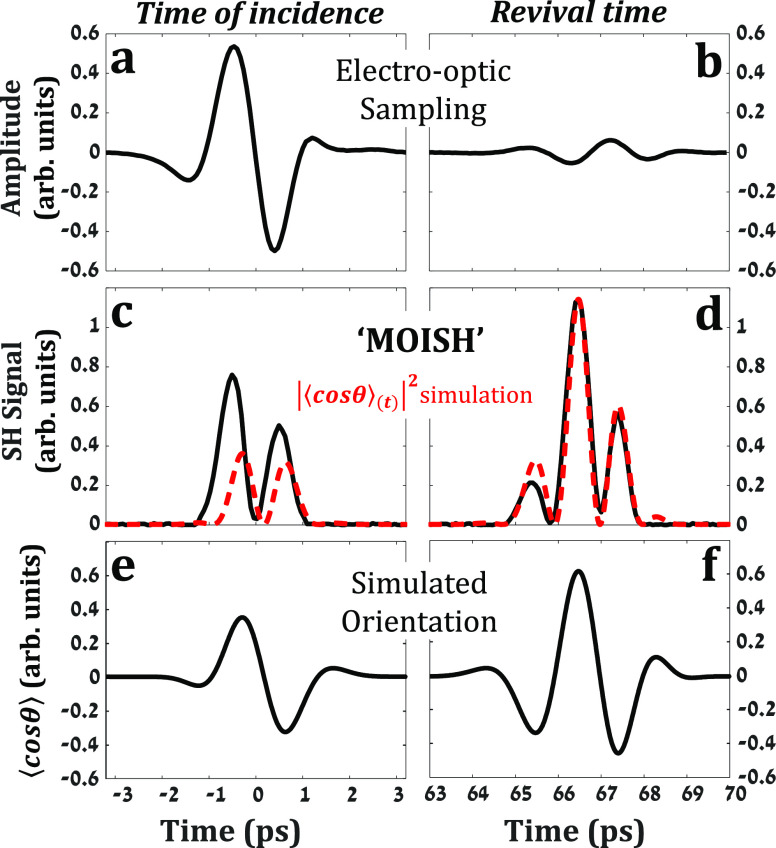
THz-induced orientation of 10 torr methyl-iodide (CH_3_I) at room temperature. (a) and (b) EOS signal at *t* = 0 and at *t* = 1*T*_rev_, respectively. (c) and (d) Second-harmonic (MOISH) signal (probe intensity 1.1 × 10^14^ W/cm^2^) at *t* = 0 and at *t* = 1*T*_rev_, respectively. (e) and (f) Simulated orientation dynamics at *t* = 0 and at *t* = 1*T*_rev_ respectively.

Figure 1 compares the experimental results obtained with EOS and MOISH from methyl-iodide (CH_3_I) gas (10 torr, 300 K) following irradiation by a single-cycle THz field generated by optical rectification in a LiNbO_3_ crystal.^39^ In EOS, the THz field propagates through the static gas cell located at the first focus of a 4-f setup.^18,19^ The THz (and succeeding FID) are recollimated and focused onto the EO detection crystal (GaP) and sampled by a weak NIR probe. Figure 1a,b shows the EOS signal with the input THz field (Figure 1a, at *t* = 0) and the FID emission at the first revival of the gas (Figure 1b, *T*_rev_ ∼ 66 ps).^31,40−42^Figure 1c,d depicts the time-resolved MOISH signal at the same respective intervals. Here, the THz field and the NIR pulse copropagate to focus at the center of the static gas cell and the generated SH signal is recorded as a function of their delay apart. While EOS detects the THz radiation (FID), MOISH is primarily sensitive to the degree of molecular orientation ⟨cos θ⟩. This is evident from the difference in signals’ amplitudes of the incident THz field (*t* = 0) and the FID emission at the revival time; In EOS, the incident THz field reaches a peak value of 0.5 while the FID emitted at *t* = *T*_rev_ remains well below 0.1 (in the arb. units shared by Figure 1a,b).

In contrast, the MOISH signal obtained at *T*_rev_ ∼ 66 ps (Figure 1d) is ∼50% larger than that of the incident THz field (Figure 1c), in good agreement with the simulated orientation dynamics ⟨cos θ⟩_(*t*)_ (Figure 1e,f). Note that the dashed red line in Figure 1c,d shows the absolute value squared |⟨cos θ⟩_(*t*)_|^2^ of the simulation results shown in Figure 1e,f because MOISH provides a homodyne signal. The maximal orientation signal at *t* = *T*_rev_ and not during, or in the vicinity of the THz excitation is an intriguing signature for the resonant nature of the THz-dipole interaction, indicating that the molecules continue to accumulate rotational energy in a coherent manner throughout the entire interaction with the field and beyond the initial event of orientation around *t* = 0. These rotational coherences manifest later on by enhanced orientation at *t* = *T*_rev_, long after the THz field is over.^8,17^ We note that while CH_3_I is a symmetric top, with rotational constants (*B* = *C* = 0.25 cm^–1^, *A* = 5.17 cm^–1^), the rotational dynamics responses of the methyl group are hindered in both THz orientation and NIR alignment experiments. In fact, previous studies have shown that the rotational dynamics of CH_3_I is fully captured when modeled as a linear rotor with *B* = 0.25 cm^–1^.^31,41^

While the MOISH signals in Figure 1c,d are qualitatively in agreement with the theoretical predictions of Figure 1e,f (dashed red lines), in what follows we focus on their quantitative discrepancies. The main disagreement is revealed when comparing the ratio of the *t* = *T*_rev_ signal (*S*_*T*_rev__) and the *t* = 0 signal (*S*_0_) given by *R*_MOISH_ = ∫ *S*_*T*_rev__d*t*/ ∫ *S*_0_d*t* to the simulated ratio *R*_Orient_^theory^ = ∫ |⟨cos θ⟩_(*T*__rev__)_|^2^d*t*/ ∫ |⟨cos θ⟩_(0)_|^2^d*t*. The latter was found to be *R*_Orient_^theory^∼2.8 and insensitive to the carrier-envelope phase (CEP, see Supporting Information SI.1, which includes refs (43, 44).) of the THz field, whereas the experimental *R*_MOISH_ in Figure 1c,d yields *R*_MOISH_ = 1.8. The discrepancy in Figure 1c of the relative peak intensities and the slight temporal shift between the experimental (black curve) and simulated results (dashed red curve) is readily observed and will become clearer in what follows.

**Figure 2:**
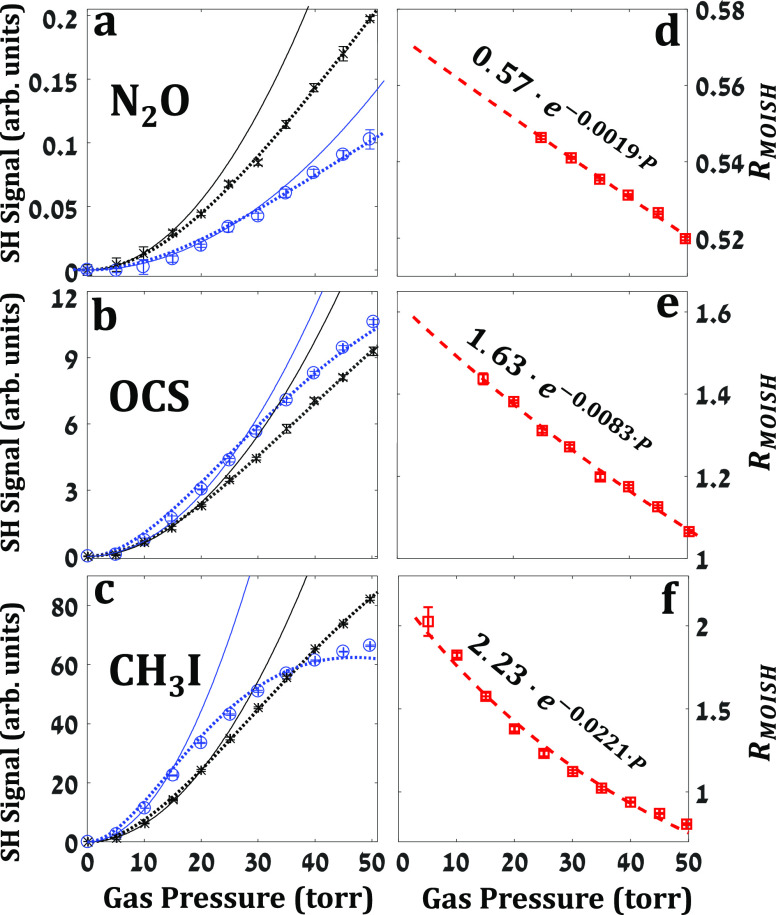
Experimental MOISH signals from different gases at varying pressures. (a–c) Show the integrated signals at *t* = 0 (black *x*’s and dotted trend-line) and at *t* = 1*T*_rev_ (blue circles and dotted trend-line) from N_2_O, OCS, and CH_3_I respectively. (d–f) Show the *R*_MOISH_ of the signals in (a–c), respectively. All measurements were performed with a probe intensity of ∼3 × 10^13^ W/cm^2^. The expected quadratic pressure dependence is depicted by the solid black and blue lines, respectively.

In a set of measurements performed with different polar gas species at varying pressures, we have found that *R*_MOISH_ varies with both the type and density of the gas, as shown in Figure 2 for three different gas species (CH_3_I, OCS, and N_2_O) in the pressure range of 0-50 torr. We note that Figures 2a–c and 4a share the same intensity scale (given in arb. units) demonstrating that the CH_3_I signal is 1–2 orders of magnitude larger than those of OCS and N_2_O. This large variation emanates from the first-order (β) and second-order (γ) hyperpolarizabilities of the different gases as predicted by our calculations (for computational details see Supporting Information SI.3 that includes refs (45−62)).

Figure 2a–c depict the SHG measured at *t* = 0 (∫*S*_0_d*t*, black data points) and *t* = *T*_rev_ (∫*S*_*T*_rev__d*t*, blue data points) for the three gas samples. The solid black and blue lines show the expected SHG dependence on pressure, produced by extrapolation of the quadratic fit of the first few (low pressure) data points. Trend-lines of the experimental data sets are given by the dotted curves. The deviation of the experimental SH from quadratic pressure dependence is attributed to:

(1) Collisional decoherence that effectively attenuates *S*_*T*_rev__ and hardly affects *S*_0_.
(2) Phase-mismatch experienced by the nonlinear SHG upon propagation in the gas.

Naturally, both these effects increase with gas density.

To ease the phase matching constraints, we restricted the interaction length by placing an iris in the gas cell.^35^ Furthermore, we note that the SHG signals at *t* = 0 and *t* = *T*_rev_ are affected similarly by phase-mismatch, thus their ratio (*R*_MOISH_) is insensitive to phase-mismatch ramifications. Figure 2d–f depict the *R*_MOISH_ obtained from the data in 2a–c, respectively. Owing to the collisional decoherence, *R*_MOISH_ decays exponentially with pressure at a specific rate for each gas and is in good agreement with the decay rates obtained via EOS (see Supporting Information SI.2) and reported in ref (18). From the fitted exponential curves in Figure 2d–f, we find that the collision-free *R*_MOISH_ values (at *P* = 0, given by the pre-exponential factors) and *R*_Orient_^theory^∼2.8 remain in discrepancy (*R*_MOISH_ = 0.57,1.63,2.23 for N_2_O, OCS, and CH_3_I, respectively). This is attributed to the TFISH signal that is induced by the incident THz field (*t* = 0) and constructively adds to the MOISH signal. The sum of these two contributions increases *S*_0_ (at the denominator of *R*_MOISH_) and results in lower *R*_MOISH_ than that expected by orientation only. In what follows, we analyze the nuclear (MOISH) and electronic (TFISH) contributions to the nonlinear susceptibility of the medium, χ^(2)^, that give rise to the observed SHG signals.

A THz field induces instantaneous inversion asymmetry as it acts on the electronic cloud of the gas molecules, forcing the electrons to oscillate in the direction of the field. This results in an effective χ_elect_^(2)^ that is detected via TFISH.^38^ Although typically performed in air or other nonpolar gas samples, the THz-induced χ_elect_^(2)^ is valid in polar molecules as well. In polar molecules however, the single-cycle THz field induces another type of inversion asymmetry as it orients the molecular dipoles^7,8^ and yields an effective χ_orient_^(2)^ that enables MOISH.

**Figure 3:**
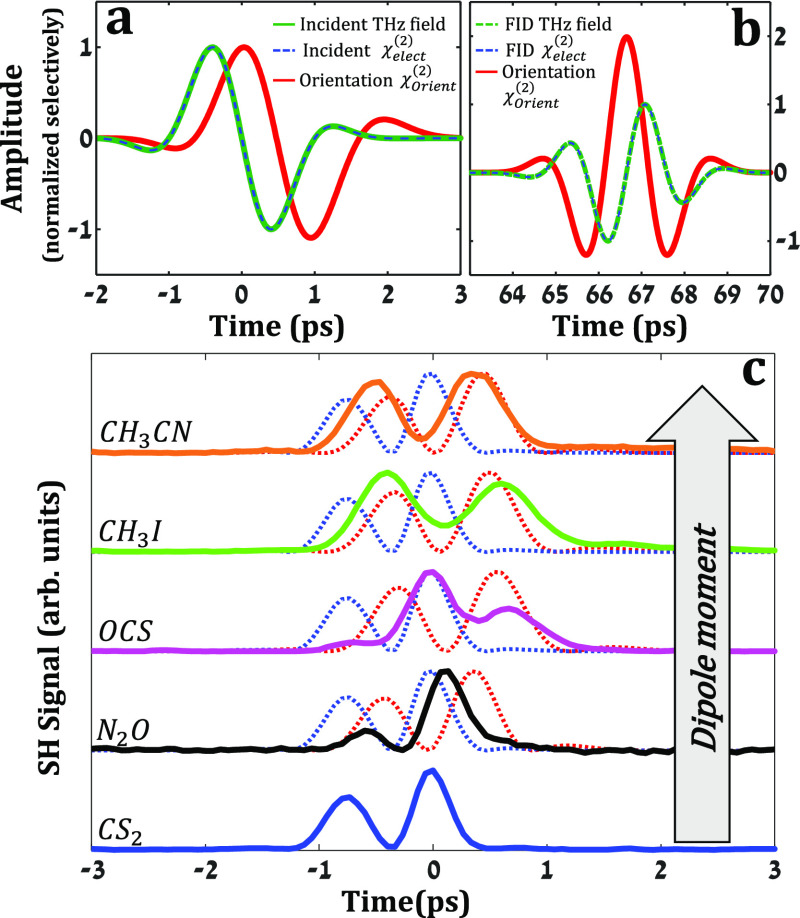
Simulation of the different χ^(2)^ contributions at (a) *t* = 0 and (b) *t* = *T*_rev_. The green curve depicts the incident THz field resulting in electronic (dashed blue curve) and the nuclear orientation (solid red curve) contributions to the nonlinear susceptibility χ^(2)^. (c) Time-resolved SHG signal at the incidence of the THz field for different gases. The electronic and nuclear contributions to χ^(2)^ are depicted by the dotted blue and red curves, respectively.

Figure 3 depicts the distinct electronic and nuclear contributions at *t* = 0 and *t* = *T*_rev_. The solid green curve in Figure 3a depicts the incident single-cycle THz field with an antisymmetric CEP, given by: *E*_THz_(*t*) =  where  and ω_0_ = 0.5 THz. The electronic contribution induced by the field is instantaneous with the latter and depicted by the dashed blue curve in Figure 3a. The solid red curve depicts the nuclear contribution χ_orient_^(2)^ of the oriented gas molecules ⟨cos θ⟩_(*t*)_. The latter was simulated by numerically propagating the density matrix ,ρ, via the Liouville–Von Neumann equation , with  and *V̂* = – μ⃗ · *E⃗*_THz_(*t*) the dipole interaction term.^8^ As can be seen in Figure 3a, the electronic and nuclear χ^(2)^ contributions induced by the incident THz field partially overlap. We note that the incident THz field and electronic response are normalized in Figure 3a. The orientation response (solid red) is normalized by its peak at *t* = *T*_rev_ (Figure 3b, solid red curve). Another electronic contribution that may interfere with the orientation at *t* = *T*_rev_ may be induced by the emitted FID field (dashed blue curve in Figure 3b). The latter is given by the time derivative of the transient orientation^29,30^ (dashed green curve in Figure 3b).

To calculate *R*_MOISH_, one needs to sum the χ_orient_^(2)^ and χ_elect_^(2)^ to obtain an effective χ^(2)^ for *t* = 0 and *t* = *T*_rev_ selectively, with χ_elect_^(2)^ ∝ *E*_THz_, γ and χ_orient_^(2)^ ∝ E_THz_, β, μ. Here, β and γ are the electronic hyper-polarizabilities at the probe frequency upon orientation and under the action of the THz field, respectively, and μ is the permanent dipole moment of the molecule. The obtained signals (in absolute value squared) are integrated over time and their ratio corresponds to *R*_MOISH_. Because the amplitude of the emitted FID is ∼10^2^ fold smaller than that of the incident field (namely, *E*_THz (0)_ ≫ *E*_THz (*T*_rev_)_^FID^ and thus χ_elect(0)_^(2)^ ≫ χ_elect(*T*_rev_)_^(2)^), the electronic contributions primarily increase the *t* = 0 signal and diminish the *R*_MOISH_ values as shown in Figure 2. The interplay between the two contributions at *t* = 0 is further manifested in the time-resolved signals of Figure 3c. Here, we extracted the incident *E*_THz_ from the pure electronic response of the (nonpolar) CS_2_ gas. The same *E*_THz_ was used to simulate the transient orientation (⟨cos θ⟩_(*t*)_) for each gas selectively (dotted red lines in each panel). The pure electronic contribution (normalized) is plotted by the dotted blue line for reference. Figure 3c shows that while the temporal signal shapes of N_2_O and OCS are admixtures of both the χ_elect_^(2)^ and χ_orient_^(2)^, the shapes of CH_3_I and CH_3_CN are primarily governed by the χ_orient_^(2)^. The contribution of the latter becomes more profound with the increase in molecular dipole N_2_O→OCS→CH3I→CH_3_CN (with μ = 0.17D,0.72D,1.62D,3.92D, respectively).

In what follows, we set to experimentally unveil the FID-induced χ_elect_^(2)^ at *t* = *T*_rev_. We start by considering the pressure-dependence of the different contributions discussed above: At *t* = 0, both the nuclear and electronic contributions are linear with the number of molecules in the interaction region: χ_orient_^(2)^_(0)_, χ_elect_^(2)^_(0)_ ∝ *P*. At *t* = *T*_rev_, however, the two contributions differ in their pressure dependencies: while χ_orient_^(2)^_(*T*_rev_)_ ∝ *P*, the FID contribution depends on the density squared χ_elect_^(2)^_(*T*_rev_)_ ∝ *P*^2^ because it is induced by the emitted FID (∝*P*) that acts back on the same gas. Furthermore, when comparing different gases, one must consider their different dipole magnitudes. Consider a THz field *E*_THz_ interacting through the molecular dipole, μ · *E*_THz_. Since the induced orientation ⟨cos θ⟩ is linear with μ, the FID that is emitted at *t* = *T*_rev_ is quadratic with μ because (18,63) where ⟨cos θ⟩ ∝ μ (for experimental *E*_FID_ vs μ see Supporting Information SI.4). From all of the above, we conclude that the extent to which the FID-induced χ_elect_^(2)^ affects the MOISH signal at *t* = *T*_rev_ depends on multiple factors: it increases with the hyperpolarizability (γ), the gas pressure ( ∝ *P*^2^), and the molecular dipole ( ∝ μ^2^).

**Figure 4:**
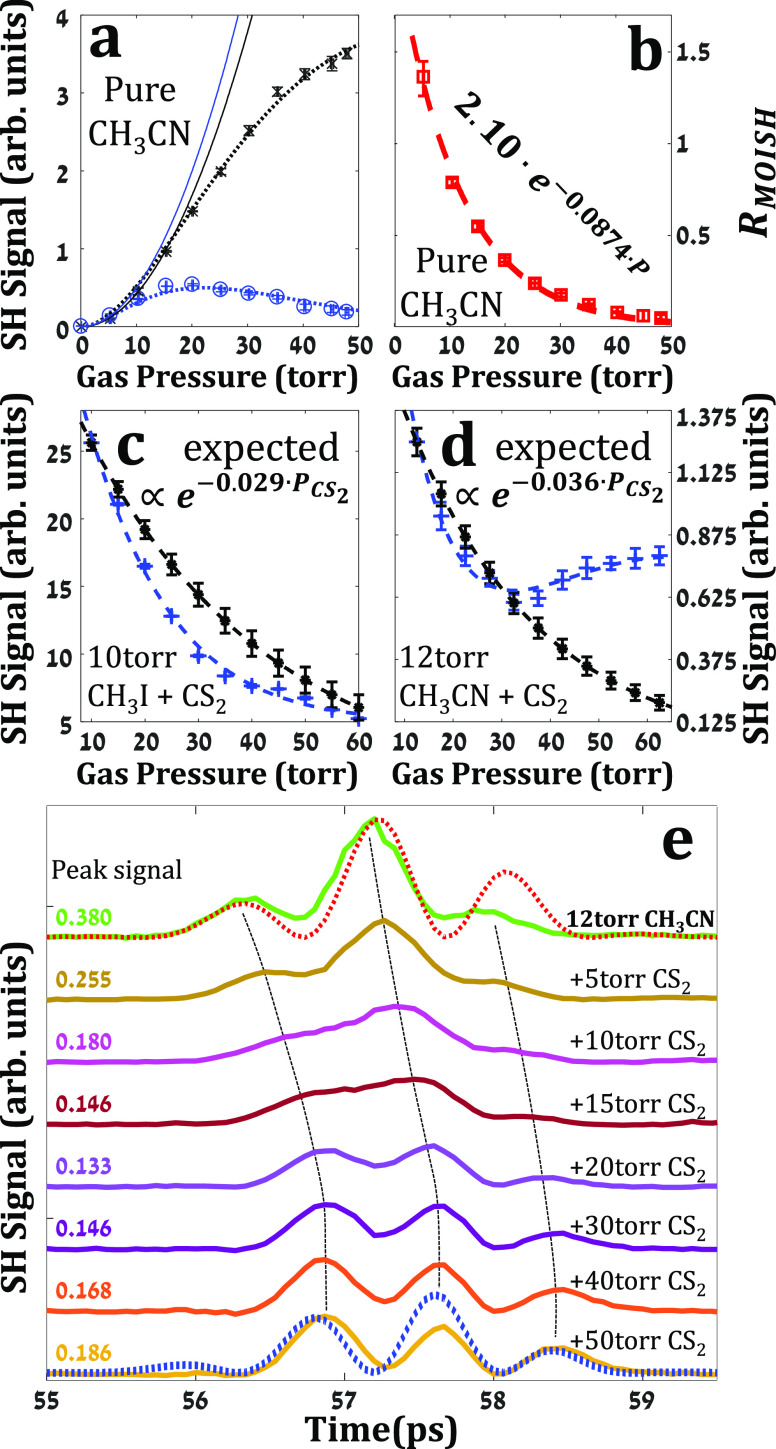
(a) and (b) Same as in Figure 2 only for CH_3_CN. (c) SH signals from a mixture of 10 torr CH_3_I and varying CS_2_ pressure. Calculated decay shown in black, and experimental decay shown in blue. (d) Same as (c) for 12 torr CH_3_CN and varying CS_2_ pressure. (e) Time-resolved signals of the data of (d) showing the evolution of the signal shape from that of MOISH (solid green) to that of FID-induced TFISH (solid yellow). Corresponding simulated results are shown by the dotted red and blue transients, respectively. The peak signal amplitudes of each transient as marked in the figure.

Thus, for gases with relatively low dipoles such as N_2_O (μ = 0.17D) and OCS (μ = 0.72D) the contribution of the FID is negligible and the decay rate of the signal at *t* = *T*_rev_ is effectively governed by collisions as shown in Figure 2a,b. For larger dipole values (such as CH_3_I with μ = 1.62D), we expect to find a larger decay rate than that induced solely from collisions. The decay rate of *R*_MOISH_ in Figure 2c however remains in good agreement with that quantified by EOS. This is attributed to the inherently large collisional decay of CH_3_I that obscures the (relatively small) contribution of the FID at *t* = *T*_rev_. Thus, a large molecular dipole acts as a “double-edged sword”—on the one hand, it increases the FID contribution but on the other hand—it enhances the collisional decay rate via dipole–dipole interactions that obscure the FID contribution. Nevertheless, an experimental indication for this elusive effect is presented in Figure 4a,b, where we conducted the exact same experiment of Figure 2 only for CH_3_CN (μ = 3.92D) and found that *R*_MOISH_ decays ∼30% faster than that quantified by EOS.

To experimentally validate the above hypothesis, one would like to vary the relative magnitudes of the two χ^(2)^ contributions selectively; however those are unavoidably inter-related. Instead, we utilized a nonpolar gas that does not contribute to χ_orient_^(2)^ but is strongly affected by the FID of the polar gas and hence serves as a “reporter gas”. This is done by injecting carbon-disulfide (CS_2_) at varying partial densities in addition to the fixed density of the polar gas (CH_3_I and CH_3_CN in Figure 4c,d, respectively). For the gas-mixing procedure, see Supporting Information SI.5 which includes ref (64).

Curves shown in Figure 4c,d were obtained with a fixed partial pressure of 10 torr CH_3_I, and 12 torr CH_3_CN, respectively, and varying CS_2_ pressures. The black curves show the expected *t* = *T*_rev_ signal of the mixture with collisional decay and phase matching effects accounted for (see Supporting Information SI.6), but without the FID contribution. The blue data points (marked by “+“) and dashed trend-lines depict the experimental results. In both gas mixtures, the FID emission interacts with the reporter CS_2_ gas and induces χ_elect_^(2)^ that partially counteracts the χ_orient_^(2)^ of the polar gas at *t* = *T*_rev_, expediting the decay of the SH signal with increased CS_2_ pressure. As the CS_2_ pressure is further increased, the decay rate of the SH gradually reduces and its trend is reversed as the orientation- and FID-induced contributions become comparable (∼30 torr in Figure 4d). Above this pressure, the SH signal starts to increase as the FID contribution overcomes that of the MOISH. This is shown in Figure 4e where the temporal shape of the signal gradually evolves from that of MOISH (from pure CH_3_CN depicted by the solid green curve) to that of the FID-induced TFISH (solid yellow), in agreement with the simulated transients (dotted red and blue, respectively). In addition, the use of the reporter, nonpolar gas, provides yet another advantage over a pure polar gas sample as the decay of *R*_MOISH_ is significantly lower in the mixtures: with 4 × 10^–3^ and 6 × 10^–3^ torr^–1^ for the CH_3_I/CS_2_ and CH_3_CN/CS_2_ mixtures compared to 2.2 × 10^–2^ and 8.7 × 10^–2^ torr^–1^ in neat gases, respectively. The reduced decay rate improves the visibility of the FID contribution that is otherwise obscured by the rapid decay rate of the pure polar gas. We further note that to alleviate possible contributions of THz-induced rotational excitation of CS_2_ owing to its large polarizability anisotropy,^21,22^ we repeated the reporter gas experiment with carbon-tetrachloride (CCl_4_) and obtained very similar trends as in Figure 4c,d.

## Conclusions

To conclude, we utilized the SH signal generated in THz-oriented gas phase molecules as a direct probe of orientation. The technique coined “MOISH“ is contributed by several electronic and nuclear (orientation) responses that temporally interfere and dictate the observed SH signal. These contributions were theoretically and experimentally explored in different gases and varying gas densities. A “reporter gas” approach was used to unveil the elusive contribution of the secondary FID emission. MOISH offers a spatially localized, all-optical technique for direct probing of molecular orientation and provides new means for studying coherent rotational dynamics induced by concerted THz and optical excitations.

## Supporting Information Available

SI.1. Theoretical dependence of R_orient_ on the carrier envelope phase of the THz field, SI.2. Experimental collisional decay rates, SI.3. Theoretical calculations of the Hyperpolarizabilities, SI.4. FID dependence on the molecular dipole, SI.5. Gas mixing procedure, and SI.6. Phase-mismatch and collisional decay of MOISH in gas mixtures (PDF)
