# Supplementary material for: Molecular Orientation-Induced Second-Harmonic Generation: Deciphering Different Contributions Apart
Source: J Phys Chem A. 2022 Jun 2;126(23):3732–8. doi: 10.1021/acs.jpca.2c03237 (PMC9207934; doi:10.1021/acs.jpca.2c03237)
Supplement: Supplementary file 1 — jp2c03237_si_001.pdf [file jp2c03237_si_001.pdf]

Molecular Orientation-Induced Second Harmonic Generation: Deciphering Different Contributions Apart

Amit Beer[1,2], Ran Damari[1,2], Yun Chen[1] and Sharly Fleischer*[1,2]
[1]Raymond and Beverly Sackler Faculty of Exact Sciences, School of Chemistry, Tel Aviv University 6997801, Israel.
[2]Tel-Aviv University center for Light-Matter-Interaction, Tel Aviv 6997801, Israel.
email address: sharlyf@tauex.tau.ac.il

Table of Contents:

The supporting information file includes the following sections:

SI.1 – Theoretical dependence of R_{orient} on the carrier envelope phase of the THz field.
SI.2 – Experimental collisional decay rates.
SI.3 – Theoretical calculations of the Hyperpolarizabilities.
SI.4 – FID dependence on the molecular dipole.
SI.5 – Gas mixing procedure.
SI.6 – Phase-mismatch and collisional decay of MOISH in gas mixtures.

SI.1) The dependence of R_{Orient}^{theory} on the CEP of the THz field

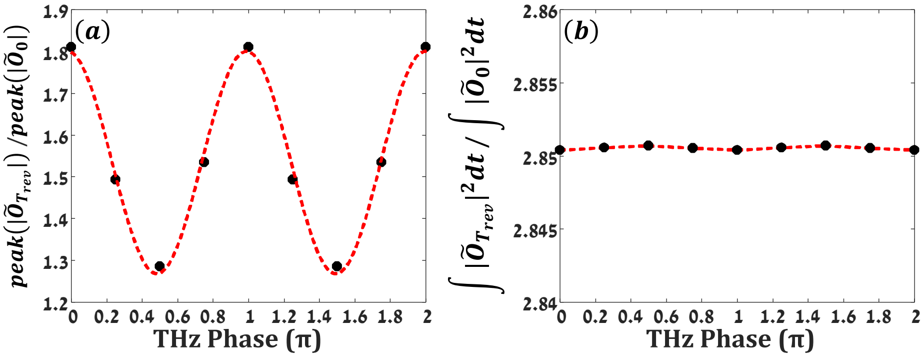

Figure S1: Simulated ratio between (a) peak orientation at t=1T_{rev} and t=0 for CH_3I for varying CEP of the incident THz field demonstrating severe dependence on the latter. (b) Calculated $R_{Orient}^{theory} = \int|\tilde{O}_{T_{rev}}|^2dt\,/\int|\tilde{O}_0|^2dt$ remains fixed at 2.85 in all CEP range.

The temporal shape of orientation depends on the carrier-envelope phase (CEP) of the THz field[1,2]. While the $\langle cos\theta\rangle_{t=0}$ shape resembles that of the incident THz field, the shape of the $\langle cos\theta\rangle_{t=T_{rev}}$ transient is π/2 shifted with respect to the latter. Thus, the ratio between the peak orientation at t=T_{rev} ($\tilde{O}_{T_{rev}}$) and at t=0 ($\tilde{O}_0$) shows strong CEP dependence and may vary between ~1.3-1.8 with CEP variations (Fig.1a). However, by taking the ratio of the integrated orientation responses i.e. $\int|\tilde{O}_{T_{rev}}|^2dt\,/\int|\tilde{O}_0|^2dt$ one alleviates the CEP dependence and obtains a fixed ratio of ~2.85 (in CH_3I)[3]. Naturally the calculated ratio of 2.85 may slightly vary with the rotational coefficient (B), the temperature and spectral width of the excitation pulse.

SI.2) Quantifying collisional decay rates via EOS measurements

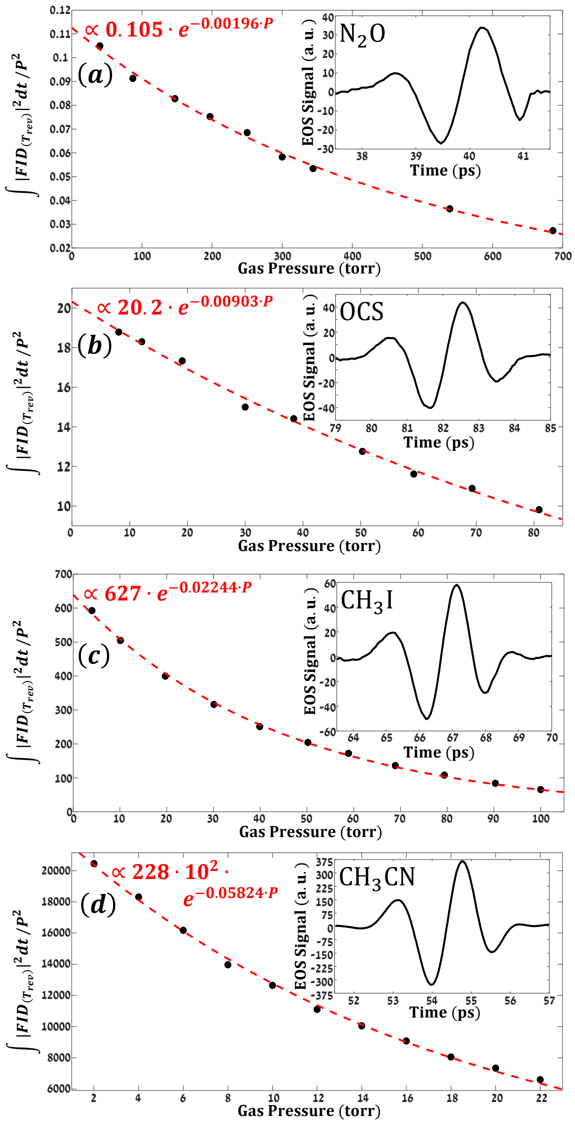

Figure S2: The pressure decay of the first FID signal (at t=1T_{rev}) for different polar gases studied in this work: (a) CH_3I, (b) OCS, (c) N_2O and (d) CH_3CN. The insets depict the typical time-resolved signal in each gas.

Figures 2d-f and Figure 4b in the main text file depict the pressure dependence of the experimental R_{MOISH} that stands for the ratio of the integrated t=0 and at t=T_{rev} signals. Since the latter is subject to collisional decay and decoherence, we set to quantify the decay rate of the molecular orientation via THz-EOS measurement. For each gas, we varied the pressure (P) and recorded the FID transients emitted at t=T_{rev} in association with the orientation of the molecules.

The FID signal amplitude increases with the gas pressure (P), and its exponential decay rate also increases linearly with P, i.e. $FID_{(T_{rev})} \propto P \cdot e^{-\gamma \cdot P \cdot t}$[4]. In order to extract the decay rates, γ [$\mathrm{sec}^{-1}\mathrm{torr}^{-1}$], of the different gasses we integrated over each transient FID signal to obtain the signal area ($\int |FID_{(T_{rev})}|^2 \mathrm{d}t$) and normalized by P^2 to obtain the data points in Figs.S2a-d. We note that this quantification metric was shown resilient to centrifugal distortion effects[3,4]. The extracted exponential decay rates were found in excellent agreement with those obtained in ref.[4] for CH_3I, OCS, N_2O and with those obtained in R_{MOISH} measurements of Fig.2a-c in the main paper file. We note that in CH_3CN however, the decay rate of R_{MOISH} (Fig.4b) is ~30% larger than in EOS (Fig.S2d). This discrepancy in CH_3CN serves as an indication for the contribution of FID to the SH signal as discussed in details in the main text.

SI.3) Theoretical calculations of Hyperpolarizabilities

The different contributions of $\chi^{(2)}_{elect}$ and $\chi^{(2)}_{orient}$ to the overall $\chi^{(2)}$ signal depends on the first (β) and second order (γ) hyperpolarizabilities respectively. These also dictate the clearly observed differences in signal amplitudes noted in Fig.2 in the main paper file. The theoretical calculations of the hyperpolarizabilities were carried out using the density functional theory. In the calculations, the molecular geometric structures were optimized at the level of B3LYP/6-311G**[5,6]. For calculating the nonlinear optical (NLO) properties of the molecules of interest we used the hybrid GGA DFT methods reported to outperform pure DFT functionals[7,8] since the fraction of the Hartree-Fock (HF) exchange in the hybrid functionals can proportionally induce a modification on the asymptotic behavior of approximate exchange-correlation (XC) potentials and a reduction in the self-interaction error[9,10]. Accordingly, the hybrid functionals B3LYP (containing 20% of exact exchange) and PBE0[11] (containing 25% of exact exchange) were chosen in this study for comparison. On the other hand, it was well known that large basis sets with polarization and diffuse functions played a significant role in a reliable estimation of the NLO properties. Herein, the diffuse function-augmented double(D)-ζ, triple(T)-ζ and quadruple(Q)-ζ basis sets aug-cc-pVnZ (n = D, T, and Q) from Dunning correlation-consistent basis set family[12–14], and the augmented triple-zeta and quadruple-zeta valence basis sets def2-nZVPD (n = T and Q) from Ahlrichs-Karlsruhe basis set family[15–18] were employed in the calculations for the first and the second hyperpolarizabilities based on their good performance on the linear and nonlinear properties of molecular materials[8,19,20].

For the first hyperpolarizability (β), the total molecular hyperpolarizability (β_{tot}) was defined as: $\beta_{tot} = \sqrt{\beta_x^2 + \beta_y^2 + \beta_z^2}$, where: $\beta_i = 1/3 \sum_j (\beta_{ijj} + \beta_{jji} + \beta_{jij})$, and where: $i, j = \{x, y, z\}$. Moreover, its projection on the dipole moment vector (β_{prj}), which was considered to be comparable to the data measured in the electric field induced second harmonic generation (EFISHG) experiment, was given by: $\beta_{prj} = \sum_i \frac{\mu_i \beta_i}{|\mu|}$. In terms of the second hyperpolarizability (γ), the total magnitude (γ_{tot}) was described as: $\gamma_{tot} = \sqrt{\gamma_x^2 + \gamma_y^2 + \gamma_z^2}$, where: $\gamma_i = 1/15 \sum_j (\gamma_{ijji} + \gamma_{ijij} + \gamma_{iijj})$. While the corresponding average magnitude $\langle\gamma\rangle$ was determined as: $\langle\gamma\rangle = \gamma_{||} = \gamma_x + \gamma_y + \gamma_z$.

All calculations were performed with Gaussian 16 package[21], while the NLO analysis was implemented by the means of Multiwfn 3.4.1[22]. The calculated results for the first and the second hyperpolarizabilities are listed in Table S1. According to the results, we can find that B3LYP and PBE0 perform similarly in the description of the NLO properties of molecules, while the results generally get converged at the level of basis sets with relatively large diffuse functions.

Table S1: The frequency-dependent first hyperpolarizability $\beta(-2\omega;\ \omega,\omega)$ ($\times$ 3.2063613061 $\cdot 10^{-53} C^3 \cdot m^3 \cdot J^{-2}$) and the second hyperpolarizability $\gamma(-2\omega;\ \omega,\omega,0)$($\times$ 6.2353799905 $\cdot 10^{-65} C^4 \cdot m^4 \cdot J^{-3}$) of CS_2, N_2O, OCS, CH_3I, and CH_3CN at wavelength of λ = 800nm calculated by B3LYP and PBE0 combined with different basis sets. In the DFT calculations, the C-S bond of CS_2 is set to be parallel to the Z direction, while for the other molecules the molecular orientations make the permanent dipole moment of these molecules point in the Z direction. For brevity, the basis sets aug-cc-pVDZ, aug-cc-pVDZ and aug-cc-pVDZ are marked as DZ, TZ, and QZ, while the prefix "def2" is omitted for the basis sets def2-TZVPD and def2-QZVPD. In addition, it should be noted that the first hyperpolarizabilities β_{tot} and β_{prj} of CS_2 are equal to 0.

**CS_2**	**B3LYP**					**PBE0**				
	**λ = 800nm**					**λ = 800nm**				
	**DZ**	**TZ**	**QZ**	**TZVPD**	**QZVPD**	**DZ**	**TZ**	**QZ**	**TZVPD**	**QZVPD**
**γ_X**	2896.9	3997.28	5060.9	4166.94	4823.15	2481.31	3556.98	4424.85	3718.26	4209.02
**γ_y**	2896.95	3997.35	5060.89	4166.87	4823.11	2481.34	3557.08	4424.85	3718.22	4209
**γ_Z**	6824.01	8384.31	9787.49	8627.42	9926.55	5841.68	7319.97	8446.47	7620.59	8604.59
**γ_{tot}**	7959.36	10112.1	12125.2	10447.9	12044.2	6814.63	8881.83	10512	9258.73	10462.8
**$\gamma_{\|\|}$**	12617.9	16378.9	19909.3	16961.2	**19572.8**	10804.3	14434	17296.2	15057.1	17022.6

**N_2O**	**B3LYP**					**PBE0**				
	**λ = 800nm**					**λ = 800nm**				
	**DZ**	**TZ**	**QZ**	**TZVPD**	**QZVPD**	**DZ**	**TZ**	**QZ**	**TZVPD**	**QZVPD**
**β_{tot}**	72.38	64.708	60.696	44.986	**52.178**	70.01	62.982	59.662	46.182	52.274
**β_{prj}**	-72.38	-64.708	-60.696	-44.986	-52.178	-70.01	-62.982	-59.662	-46.182	-52.274
**γ_X**	344.416	428.796	520.361	309.923	390.231	311.712	395.387	474.556	293.393	362.682
**γ_y**	344.416	428.796	520.361	309.923	390.231	311.712	395.387	474.556	293.393	362.682
**γ_Z**	856.978	964.236	1052.89	680.182	837.465	777.555	882.184	954.301	637.85	773.993
**γ_{tot}**	985.726	1139.07	1284.57	809.168	1002.95	893.824	1044.47	1166.66	760.928	928.516
**$\gamma_{\|\|}$**	1545.81	1821.83	2093.61	1300.03	**1617.93**	1400.98	1672.96	1903.41	1224.64	1499.36

OCS	B3LYP λ = 800nm					PBE0 λ = 800nm				
	DZ	TZ	QZ	TZVPD	QZVPD	DZ	TZ	QZ	TZVPD	QZVPD
β_{tot}	160.587	193.837	206.763	201.84	**212.191**	149.6	179.531	190.686	185.751	195.708
β_{prj}	-160.584	-193.837	-206.762	-201.839	-212.191	-149.6	-179.53	-190.686	-185.751	-195.708
γ_X	1500.49	2108.2	2709.82	2202.14	2556.46	1320.46	1912.17	2416.01	1994.74	2268.23
γ_y	1498.91	2107.81	2710.62	2203.62	2558.02	1318.56	1911.99	2417.1	1995.9	2269.39
γ_Z	1533.34	2056.23	2451.71	2149.26	2465.45	1373.46	1852.33	2178.86	1931.32	2195.29
γ_{tot}	2617.12	3621.53	4549.89	3784.8	4376.92	2317.03	3277.69	4053.01	3419.44	3887.71
$\gamma_{\|\|}$	4532.74	6272.24	7872.15	6555.02	**7579.93**	4012.48	5676.48	7011.97	5921.96	6732.91

CH_3CN	B3LYP λ = 800nm					PBE0 λ = 800nm				
	DZ	TZ	QZ	TZVPD	QZVPD	DZ	TZ	QZ	TZVPD	QZVPD
β_{tot}	14.233	25.911	30.97	6.996	**20.742**	15.545	24.115	27.929	7.793	19.401
β_{prj}	14.233	25.911	30.97	6.996	20.742	15.545	24.115	27.929	7.793	19.401
γ_X	1011.22	1235.68	1363.55	985.433	1204.01	945.547	1139.95	1248.53	929.275	1114.13
γ_y	1011.13	1235.81	1364.43	985.475	1204.03	945.471	1139.84	1248.39	929.193	1114
γ_Z	2255.52	2481.31	2593.5	2030.16	2303.92	2075.32	2266.35	2357.55	1887.86	2120.81
γ_{tot}	2670.64	3034.97	3232.21	2462.48	2864.85	2468.79	2781.2	2945.39	2300.21	2641.99
$\gamma_{\|\|}$	4277.87	4952.8	5321.48	4001.07	**4711.96**	3966.33	4546.14	4854.47	3746.32	4348.95

CH_3I	B3LYP λ = 800nm		PBE0 λ = 800nm	
	TZVPD	QZVPD	TZVPD	QZVPD
β_{tot}	361.347	**376.31**	331.104	338.647
β_{prj}	361.347	376.31	331.104	338.647
γ_X	6890.73	8589.32	6255.76	7496.88
γ_y	6890.67	8588.62	6254.16	7493.97
γ_Z	6507.53	7452.54	5917.21	6655.75
γ_{tot}	11718	14250.7	10642.5	12516.5
$\gamma_{\|\|}$	20288.9	**24630.5**	18427.1	21646.6

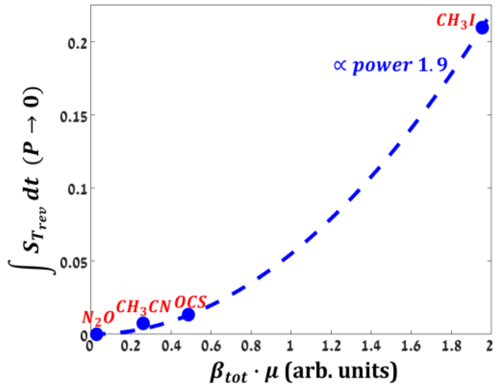

Figure S3: the figure depicts $\int S_{T_{rev}} dt$ extrapolated to $P = 0$ for the four polar gases plotted vs. $\chi^{(2)}_{orient-effective}$.

Figure S3 depicts the experimental SHG signals obtained in Figs.2a-c and Fig.4.a in the main text, at t=T_{rev} ($\int S_{T_{rev}}$dt) extrapolated to $P = 0$, i.e. under decay and decoherence free conditions, for the four polar gasses vs. the calculated hyperpolarizabilities noted above. In order to compare the experimental results of the different gasses we must consider the degree of orientation induced by the THz field. The latter is linear with the permanent dipole of the molecules. Thus, the expected SHG signal contributed by the dipole orientation must be factored by the molecular dipole: $\chi^{(2)}_{orient-effective} \propto \mu \cdot \beta$, while the electronic contribution: $\chi^{(2)}_{electronic} \propto \gamma$.

Figure S3 show that $\int S_{T_{rev}} dt \sim \left(\chi^{(2)}_{orient-effective}\right)^{1.9}$ in agreement with the expected quadratic dependence. At t=0 (not shown here) the signal includes both the nuclear and electronic contributions, that cannot be separated and therefore cannot be fitted selectively. These SH signal magnitude of the four polar gases (Figs.2a-c and Fig.4.a in the main text): $SH_{(CH_3I)} \gg SH_{(OCS)} > SH_{(N_2O)} > SH_{(CH_3CN)}$, is in good correspondence with the calculated β.

SI.4) FID dependence on the molecular dipole

In what follows we provide experimental verification for the quadratic dependence of the FID field on the molecular dipole ($E_{FID} \propto \mu^2$) using the pressure dependent FID transients measured via THz-EOS (SI.2). Consider the pre-exponential factors of the fits in Fig.S2. In fact, these pre-factors represent the integrated intensity per torr^2 $\int |E_{FID}|^2 \, dt/P^2$ of the FID, extrapolated to $P = 0$, i.e. under decay and decoherence free conditions. Since all four gas samples were measured under the exact same experimental conditions (Incident THz amplitude, temperature, interaction volume), their ratio corresponds to the ratio of the emitted energies. Thus, by plotting the extracted pre-exponential factors of Fig.S2 vs. the dipole and fitting to a power law, one obtains $\propto \mu^{4.07}$ power dependence which corresponds to quadratic dependence of E_{FID} on the molecular dipole ($E_{FID} \propto \mu^2$). To further verify the quartic power dependence that relies on 4 data points (4 gas types studied), we note (and show in the inset) that the fit results for the first three gasses yielded the same power law dependence.

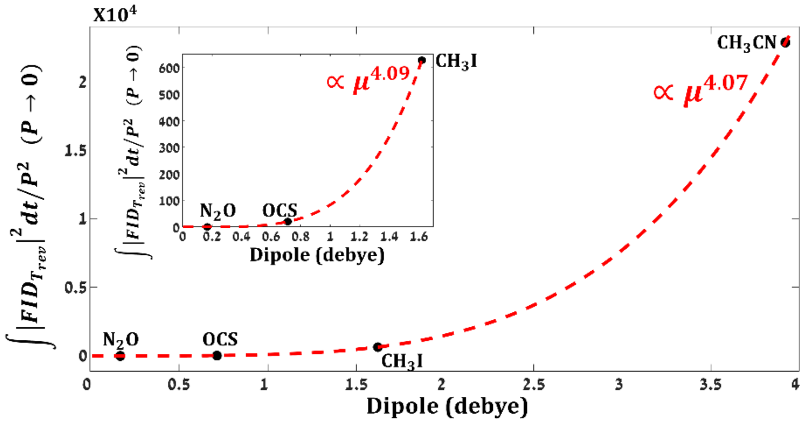

Figure S4: Extrapolated collision-free FID energy vs. the molecular dipole, showing $\propto \mu^4$ power law dependence.

SI.5) Gas mixing procedure

Gas mixtures with predetermined partial pressures of polar (CH_3I or CH_3CN) and non-polar (CS_2) "reporter gas" were prepared via the following procedure using the experimental system shown in Fig.S5. The setup includes a gas manifold connected to our static gas cell modified to include a 'cold finger'.

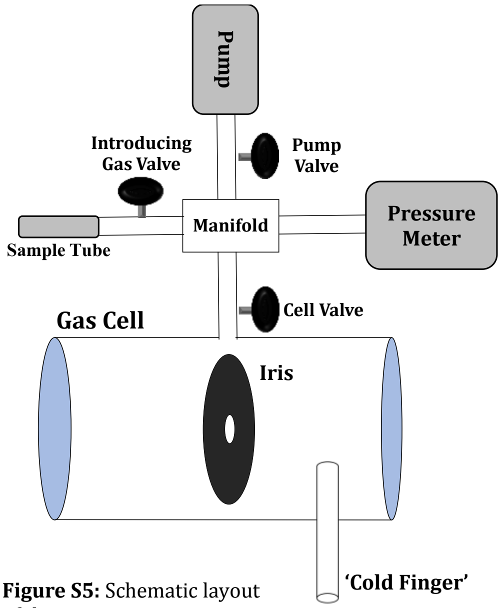

Figure S5: Schematic layout of the gas mixing setup.

First, the cell was evacuated by the vacuum pump. Then, a fixed pressure of the polar gas (10torr for CH_3I or 12torr for CH_3CN) was introduced into the entire manifold via the sample input port equipped with a Wilson seal[23]. Next, the cell valve was closed and the 'cold finger' cooled down by liquid nitrogen to condense the gas at the 'cold finger'. The manifold was evacuated down to <<0.1 torr. A small amount of CS_2 was introduced to the manifold and the manifold pressure recorded. Using our pre-calibrated ratio of the manifold volume and the cell volume, we can determine the exact pressure required in the manifold in order to achieve the desired pressure in the gas cell. By opening the cell valve we let the reporter gas condensate at the 'cold finger' until the pressure gauge reads '0' (namely <<0.1torr in our case). We close the cell valve and remove the

liquid nitrogen from the cold finger, allowing it to thermalize and the gasses to homogeneously evaporate in the closed gas cell.

SI.6) Phase-mismatch and collisional decay of MOISH in gas mixtures

Figures 4c,d in the main text compare the expected MOISH signals to the experimentally measured signals in the gas mixtures. In what follows we describe how we produced the expected curve (dashed black and data points).

The expected curve uses the SH signal measured in the pure polar gas at the fixed pressure used in the mixture, namely 10torr for CH_3I and 12torr for CH_3CN. These 'first' data points already suffer from collisional decay and phase mismatch of the pure polar gas. Thus, our task is to quantify the ramifications of the added CS_2 imparted on the SH signal within the mixture. These are the collisional decay rate γ_{CS_2} and the phase mismatch Δk_{CS_2}.

a) Quantifying collisional decay rate of the bimolecular mixture:

The collisional decay rate induced by CS_2 to the rotational dynamics of the polar gas was measured via EOS in the binary gas mixtures of interest (CH_3I and CS_2, CH_3CN and CS_2). The FID field emanates solely from the polar gas which is kept at fixed density. Therefore, by monitoring the FID field under varying CS_2 pressures, one directly obtains the CS_2-induced decay rate of interest.

Figures S6a,b show the extracted CS_2-induced decay of the orientation (FID) of CH_3I ($\gamma_{CS_2} = 4.075 \cdot 10^{-3}$ torr^{-1}and CH_3CN ($\gamma_{CS_2} = 5.74 \cdot 10^{-3}$ torr^{-1}) respectively. We note that as in section SI.2, also here we integrate over the square of the FID field and fit to exponential decay as noted in the figures.

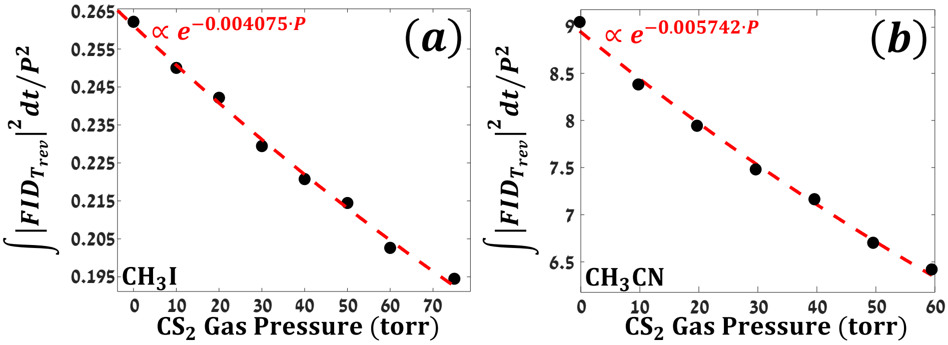

Figure S6ab: Figures a and b depict the EOS measurements done in gas-mixture of 10torr CH_3I/12torr CH_3CN and with varying CS_2 pressures respectively.

An important advantage of the 'reporter gas' approach is the reduced decay rate imposed by CS_2 on the polar gas dynamics compared to the decay rate of pure polar gasses: while the pure polar gasses decay with $\gamma^{CH_3I} \sim 0.022 \text{torr}^{-1}$ and $\gamma^{CH_3CN} \sim 0.058 \text{torr}^{-1}$ due to strong dipole-dipole interactions, they decay with $\gamma_{CS_2}^{CH_3I} \sim 0.004 \text{torr}^{-1}$ and $\gamma_{CS_2}^{CH_3CN} \sim 0.006 \text{torr}^{-1}$. The drastically reduced decay rate is yet another advantage that increases the visibility of the FID contribution to the SH signal via the "reporter-gas" technique.

b) Quantifying the CS_2-induced phase mismatch (Δk)

The expected curves of Fig.4c,d in the main text use the experimentally measured signal of the pure polar gas as the basis of the SH signal at t=T_{rev} and account for the prospected decay of the signal due to collisions with the added CS_2 (S6a,b) and the phase mismatch it imposes as described in this section. In fact, the phase mismatch introduced by 10torr of CH_3I or 12torr CH_3CN are inherently included in the first data point of each curve, thus our mission reduces to extract the Δk contributed by CS_2 at the varying pressures. For this task we performed a set of TFISH measurements in neat CS_2 gas at varying pressures.

The SH signal from the nonpolar CS_2 gas depends on the gas pressure: $E_{SH} \propto P_{CS_2} \cdot e^{-\Delta k_{CS_2} \cdot L \cdot P_{CS_2}}$

Namely, the emitted SH field is proportional to the gas pressure. The phase-mismatch $e^{-\Delta k_{CS_2} \cdot L \cdot P_{CS_2}}$ also increases with pressure (here Δk_{CS_2} is given in [$\mathrm{cm}^{-1} \cdot \mathrm{torr}^{-1}$] and L is the effective length of interaction). By fitting our experimental results to a set of TFISH measurements at varying CS_2 pressures we can extract Δk_{CS_2}. Specifically, since we measure the SH intensity $|E_{SH}|^2$, we fit our data points to $I_{SH} \propto {P_{CS_2}}^2 \cdot e^{-2 \cdot \Delta k_{CS_2} \cdot L \cdot P_{CS_2}}$ as shown in Figs. S6c,d.

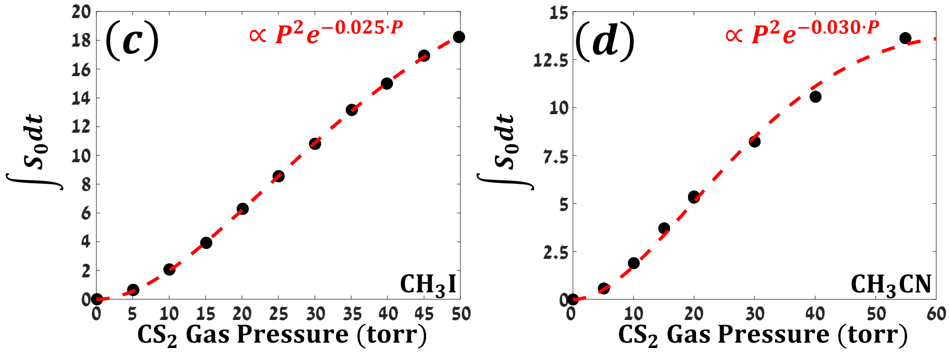

Figure S6cd: Figures c and d show SH measurements done in pure CS_2 gas sample with the same experimental configurations of their corresponding figures 4c,d in the main paper file.

Figures S6c,d show the integrated TFISH signals of pure CS_2 gas at varying pressures. It may seem peculiar that these two, allegedly similar, data sets provide slightly different phase-mismatch parameters. However, we remind the readers that our experimental gas cell is equipped with an iris that serves to ease the phase matching constraints as reported previously[24]. This iris effectively reduces the interaction length by blocking the THz beam around the focus of the optical probe. The exact position of the iris is determined by the optimal (maximal) MOISH signal of the pure polar sample (CH_3I and CH_3CN in c,d respectively). Once the iris is set in position, we evacuate the cell and perform the CS_2 TFISH measurements, keeping the iris position fixed throughout all measurements of the pure and mixed samples. Since the exact iris position may be slightly differ for each sample, the retrieved phase mismatch of the CS_2 may slightly differ with different samples as shown by the experimental results.

With both the decay rate of the mixtures extracted from EOS measurements (Fig.S6ab) and the phase mismatch (for specific iris configuration) extracted from TFISH measurements of the CS_2 reporter gas in hand, we can calculate and plot the expected SH signal as noted in the following table:

Gas sample	Measured SH $\int S_{T_{rev}} dt$ (arb. units)	Fitted phase-mismatch $2\Delta k_{CS_2} \cdot L$ (torr^{-1})	Collision decay $2\gamma_{mixture} \cdot T_{rev}$ (torr^{-1})	Expected signal
CH_3I (10torr)	25.6	0.02492	0.004075	$\mathbf{25.6 \cdot e^{-0.029 \cdot P_{CS_2}}}$
CH_3CN (12torr)	1.25	0.03044	0.005742	$\mathbf{1.25 \cdot e^{-0.036 \cdot P_{CS_2}}}$

Table S2: Phase-matching and collisional decay rates extracted from Figs.S6.

List of References:

(1) Ahmed, S.; Savolainen, J.; Hamm, P. The Effect of the Gouy Phase in Optical-Pump-THz-Probe Spectroscopy. *Opt. Express* **2014**, *22* (4), 4256.

(2) Fleischer, S.; Zhou, Y.; Field, R. W.; Nelson, K. A. Molecular Orientation and Alignment by Intense Single-Cycle THz Pulses. *Phys. Rev. Lett.* **2011**, *107* (16), 163603.

(3) Owschimikow, N.; Königsmann, F.; Maurer, J.; Giese, P.; Ott, A.; Schmidt, B.; Schwentner, N. Cross Sections for Rotational Decoherence of Perturbed Nitrogen Measured via Decay of Laser-Induced Alignment. *J. Chem. Phys.* **2010**, *133* (4), 044311.

(4) Damari, R.; Rosenberg, D.; Fleischer, S. Coherent Radiative Decay of Molecular Rotations: A Comparative Study of Terahertz-Oriented versus Optically Aligned Molecular Ensembles. *Phys. Rev. Lett.* **2017**, *119* (3), 033002.

(5) Becke, A. D. A New Mixing of Hartree–Fock and Local Density-functional Theories. *J. Chem. Phys.* **1993**, *98* (2), 1372–1377.

(6) Krishnan, R.; Binkley, J. S.; Seeger, R.; Pople, J. A. Self-consistent Molecular Orbital Methods. XX. A Basis Set for Correlated Wave Functions. *J. Chem. Phys.* **1980**, *72* (1), 650–654.

(7) Champagne, B.; Perpète, E. A.; Jacquemin, D.; Van Gisbergen, S. J. A.; Baerends, E. J.; Soubra-Ghaoui, C.; Robins, K. A.; Kirtman, B. Assessment of Conventional Density Functional Schemes for Computing the Dipole Moment and (Hyper)Polarizabilities of Push-Pull π-Conjugated Systems. *J. Phys. Chem. A* **2000**, *104* (20), 4755–4763.
(8) Sałek, P.; Helgaker, T.; Vahtras, O.; Ågren, H.; Jonsson, D.; Gauss, J. A Comparison of Density-Functional-Theory and Coupled-Cluster Frequency-Dependent Polarizabilities and Hyperpolarizabilities. *Mol. Phys.* **2005**, *103* (2–3), 439–450.
(9) Autschbach, J.; Srebro, M. Delocalization Error and "Functional Tuning" in Kohn-Sham Calculations of Molecular Properties. *Acc. Chem. Res.* **2014**, *47* (8), 2592–2602.
(10) Vydrov, O. A.; Scuseria, G. E.; Perdew, J. P.; Ruzsinszky, A.; Csonka, G. I. Scaling down the Perdew-Zunger Self-Interaction Correction in Many-Electron Regions. *J. Chem. Phys.* **2006**, *124* (9) , 094108.
(11) Adamo, C.; Barone, V. Toward Reliable Density Functional Methods without Adjustable Parameters: The PBE0 Model. *J. Chem. Phys.* **1999**, *110* (13), 6158–6170.
(12) Dunning, T. H. Gaussian Basis Sets for Use in Correlated Molecular Calculations. I. The Atoms Boron through Neon and Hydrogen. *J. Chem. Phys.* **1989**, *90* (2), 1007–1023.
(13) Kendall, R. A.; Dunning, T. H.; Harrison, R. J. Electron Affinities of the First-Row Atoms Revisited. Systematic Basis Sets and Wave Functions. *J. Chem. Phys.* **1992**, *96* (9), 6796–6806.
(14) Woon, D. E.; Dunning, T. H. Gaussian Basis Sets for Use in Correlated Molecular Calculations. III. The Atoms Aluminum through Argon. *J. Chem. Phys.* **1993**, *98* (2), 1358–1371.
(15) Peterson, K. A.; Figgen, D.; Goll, E.; Stoll, H.; Dolg, M. Systematically Convergent Basis Sets with Relativistic Pseudopotentials. II. Small-Core Pseudopotentials and Correlation Consistent Basis Sets for the Post-d Group 16-18 Elements. *J. Chem. Phys.* **2003**, *119* (21), 11113–11123.
(16) Weigend, F.; Ahlrichs, R. Balanced Basis Sets of Split Valence, Triple Zeta Valence and Quadruple Zeta Valence Quality for H to Rn: Design and Assessment of Accuracy. *Phys. Chem. Chem. Phys.* **2005**, *7* (18), 3297–3305.
(17) Rappoport, D.; Furche, F. Property-Optimized Gaussian Basis Sets for Molecular Response Calculations. *J. Chem. Phys.* **2010**, *133* (13), 134105.
(18) Weigend, F.; Furche, F.; Ahlrichs, R. Gaussian Basis Sets of Quadruple Zeta Valence Quality for Atoms H-Kr. *J. Chem. Phys.* **2003**, *119* (24), 12753–12762.
(19) Hickey, A. L.; Rowley, C. N. Benchmarking Quantum Chemical Methods for the Calculation of Molecular Dipole Moments and Polarizabilities. *J. Phys. Chem. A* **2014**, *118* (20), 3678–3687.
(20) Zalešny, R.; Baranowska-Ączkowska, A.; Medveŋ, M.; Luis, J. M. Comparison of Property-Oriented Basis Sets for the Computation of Electronic and Nuclear Relaxation Hyperpolarizabilities. *J. Chem. Theory Comput.* **2015**, *11* (9), 4119–4128.
(21) Frisch, M. J. *et.al*, G16_C01. 2016, p Gaussian 16, Revision C.01, Gaussian, Inc., Wallin.
(22) Lu, T.; Chen, F. Multiwfn: A Multifunctional Wavefunction Analyzer. *J. Comput. Chem.* **2012**, *33* (5), 580–592.
(23) Wilson, R. R. A Vacuum-Tight Sliding Seal. *Rev. Sci. Instrum.* **1941**, *12* (2), 91–93.
(24) Beer, A.; Hershkovitz, D.; Fleischer, S. Iris-Assisted Terahertz Field-Induced Second-Harmonic Generation in Air. *Opt. Lett.* **2019**, *44* (21), 5190.
